# Supplementary material for: A novel circulating tamiami mammarenavirus shows potential for zoonotic spillover
Source: PLoS Negl Trop Dis. 2020 Dec 28;14(12):e0009004. doi: 10.1371/journal.pntd.0009004 (PMC7794035; doi:10.1371/journal.pntd.0009004)
Supplement: S1 Text — (DOCX) [file pntd.0009004.s001.docx]

**Text S1. TAMV-Ref and TAMV-FL similarly bind heparan sulfate proteoglycans.**

Because of the high number of amino acid substitutions between TAMV-Ref and TAMV-FL GP we compared their ability to bind HSPG. For that aim, we performed TAMV-Ref and TAMV-FL PV along with EBOV PV infections in A549 cells in presence of heparin, which competes with HSPG for GP interaction. EBOV PV is dramatically inhibited, even at the lowest heparin concentration, while both TAMV-Ref and TAMV-FL exhibits limited susceptibility to inhibition by heparin (Fig. S5A). To validate these results, we also performed PV infections in A549 cells treated with heparinase III, which cleaves and removes the polysaccharide chains from the HSPG protein component (Fig. S5B). The results confirm that both TAMV-Ref and TAMV-FL exhibit comparable HSPG binding affinities.
