# Supplementary material for: A novel circulating tamiami mammarenavirus shows potential for zoonotic spillover
Source: PLoS Negl Trop Dis. 2020 Dec 28;14(12):e0009004. doi: 10.1371/journal.pntd.0009004 (PMC7794035; doi:10.1371/journal.pntd.0009004)
Supplement: S2 Text — (DOCX) [file pntd.0009004.s002.docx]

**Text S2. TAMV-Ref GP but not TAMV-FL GP induce syncytia formation at neutral pH, and it is increased by D156N mutation.**

The fusogenic activity of mammarenavirus GP at low pH has been extensively documented (1-3). Intriguingly, despite the lack of reports about mammarenavirus GP-mediated fusion activity at neutral pH, we observe syncytia formation in TAMV-Ref GP transfected cells. To further investigate this finding, we used a NanoLuc protein-fragment assay (4) to obtain a quantitative assessment of the fusion activity of the different mammarenavirus GPs (Fig. S6A). To this end, 293T were co-transfected with plasmids encoding for mammarenaviral GPs and required reporter plasmids for the NanoLuc protein-fragment assay, and monitored syncytia formation (Figs. S6B, C). Among all tested GP, only TAMV-Ref GP induces syncytia. Furthermore, neither N151K, D156N nor both mutations together, lead to any increase of syncytia formation of TAMV-FL (Fig. S6A-B). Despite no effect when inserted in TAMV-FL GP, D156N substitution in TAMV-Ref GP, but not N151K or both mutations together, dramatically increases the intrinsic syncytia formation ability of TAMV-Ref. In all cases, cell death occurs upon syncytia formation (48h after transfection).

**SUPPLEMENTAL MATERIAL AND METHODS.**

**Syncytia formation**. For monitoring syncytia formation upon arenavirus GP transfection, we followed the scheme on Fig. S3A. 293T cells were cotransfected with HiBit and GP-expressing plasmids and with LgBit and GP-expressing plasmids (10 µg total DNA per 10 cm dishes) and incubated for 6h at 37°C and 5% (vol/vol) CO_2_. After incubation, cells were plated in poly-lysine-treated 96-well plate (suitable for luminescence measurement). 24 hours after transfection, syncytia formation was scored using an EVOS Floid Cell Imaging Station 20X Plan fluorite lens (Thermo Fisher Scientific, Waltham, Massachusetts, USA). Samples were also assayed for luciferase activity with Nano-Glo live cell assay system from Promega (Madison, Wisconsin, USA), following instructions from the manufacturer.

**REFERENCES**:

1. Castilla V, Mersich SE, Candurra NA, Damonte EB. The Entry of Junin Virus into Vero Cells. Arch Virol. 1994;136(3-4):363-74.

2. Castilla V, Contigiani M, Mersich SE. Inhibition of cell fusion in Junin virus-infected cells by sera from Argentine hemorrhagic fever patients. J Clin Virol. 2005;32(4):286-8.

3. York J, Nunberg JH. Intersubunit interactions modulate pH-induced activation of membrane fusion by the Junin virus envelope glycoprotein GPC. J Virol. 2009;83(9):4121-6.

4. Torriani G, Trofimenko E, Mayor J, Fedeli C, Moreno H, Michel S, et al. Identification of clotrimazole-derivatives as specific inhibitors of Arenavirus fusion. J Virol. 2019.
