# Supplementary figures and images for: A novel circulating tamiami mammarenavirus shows potential for zoonotic spillover
Source: PLoS Negl Trop Dis. 2020 Dec 28;14(12):e0009004. doi: 10.1371/journal.pntd.0009004 (PMC7794035; doi:10.1371/journal.pntd.0009004)

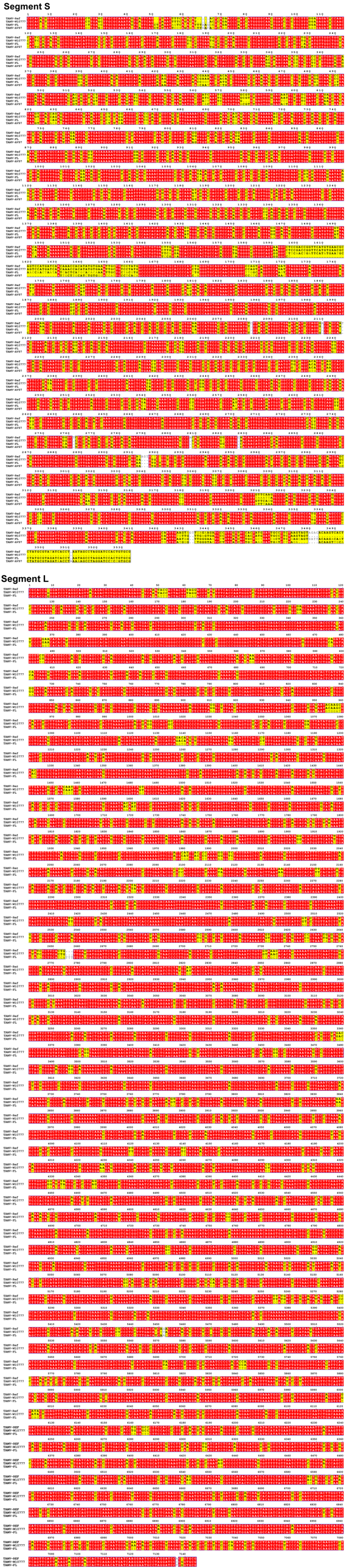

Supplement: S1 Fig — For S segment, TAMV-Ref sequence [47], TAMV W10777 [49], TAMV-FL (MK500936) and TAMV AV97140103 (Abbreviated as TAMV-AV97; EU486821.1) were used. For L segment TAMV-Ref sequence [47], TAMV W10777 [49], and TAMV-FL (MK500937). SNVs are highlighted with yellow boxes, and bold letters are used for the most frequent base at each position. (TIF) [file pntd.0009004.s003.tif]

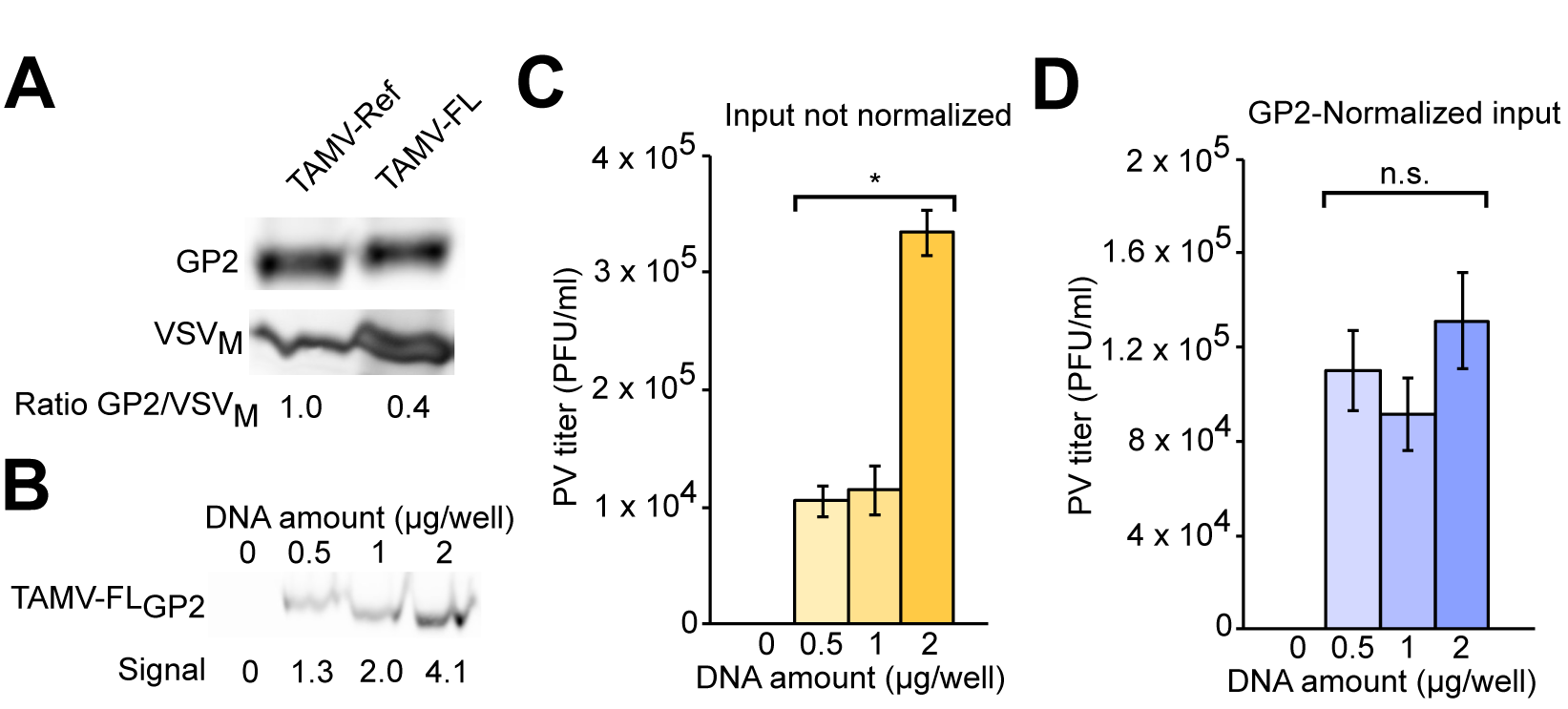

Supplement: S2 Fig — (A) TAMV-Ref and TAMV-FL PV were purified by ultracentrifugation in 30% sucrose cushion (3h, 100,000 x g, 4°C), lysed, separated by SDS-PAGE and assayed for TAMV-FL GP2 detection (anti-HA monoclonal antibody) and VSVM (specific VSV-M antibody) in immunoblotting. (B) TAMV-FL PV were produced by transfecting indicated amounts of TAMV-FLGP DNA. Obtained PV preparations were purified by ultracentrifugation in 30% sucrose cushion (3h, 100000 x g, 4°C), lysed, separated by SDS-PAGE and assayed for TAMV-FL GP2 detection (anti-HA monoclonal antibody) by immunoblotting. (C) A549 cells were infected with equal volumes of TAMV-FL preparations produced in (B). (D) A549 cells were infected with amounts of TAMV-FL normalized with obtained GP-signal in (B). Error bars in panels (C) and (D) represent standard deviations (n = 3). Asterisks in panels (C) and (D) denote statistical significance in ANOVA test (ns: p>0.05; *: p<0.05). (TIF) [file pntd.0009004.s004.tif]

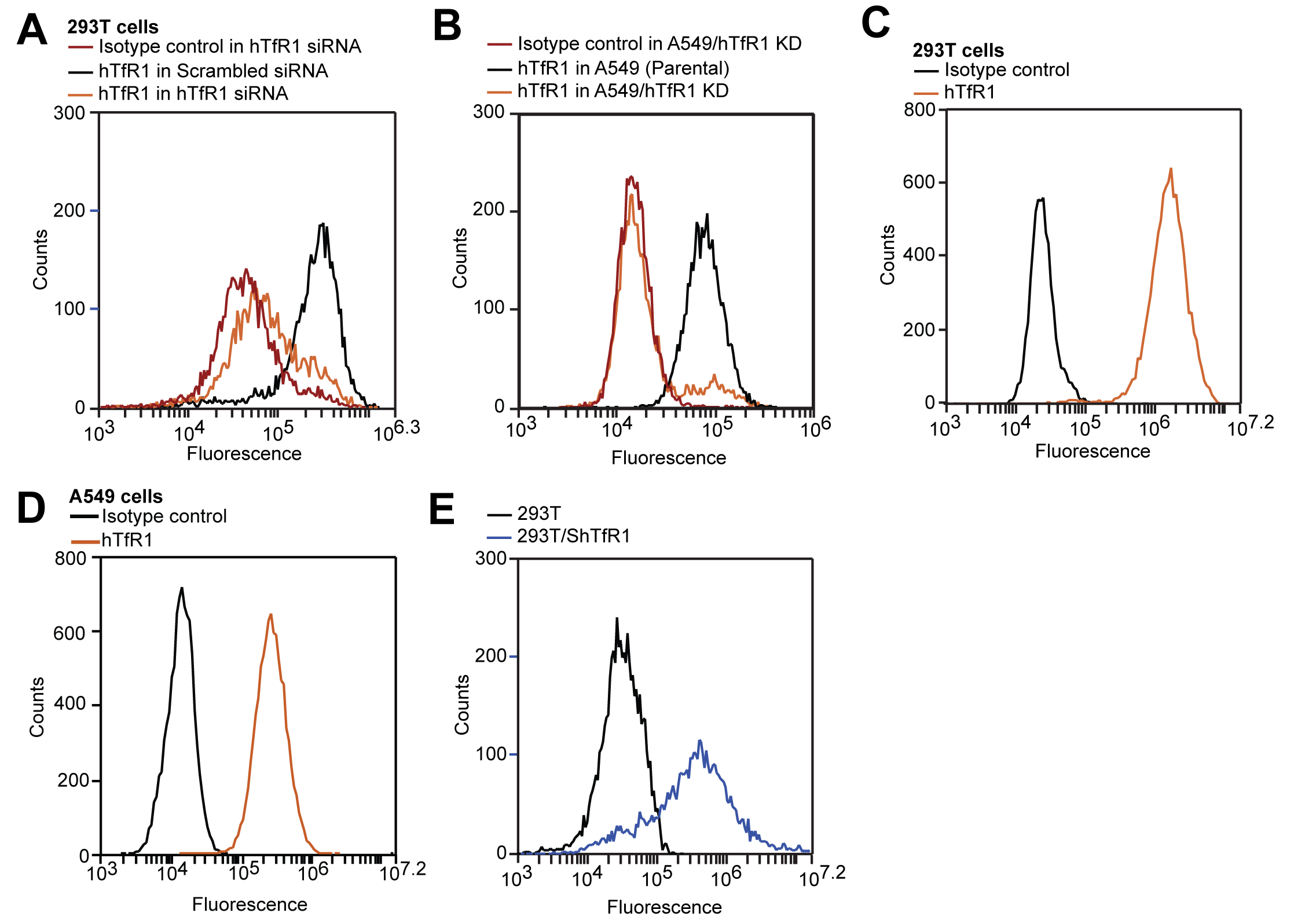

Supplement: S3 Fig — (A) Endogenous hTfR1 expression in siRNA hTfR1-transfected 293T cells. Non-targeted probe (scrambled siRNA) was used as control. Receptor expression was assessed by specific hTfR1 (CD71) monoclonal antibody or respective isotype control. (B) Endogenous hTfR1 expression in A549/hTfR1 KD cells obtained by CRISPR/Cas9. Non-transduced parental A549 cells were used as control. Receptor expression was assessed by specific hTfR1 (CD71) monoclonal antibody or respective isotype control. Endogenous hTfR1 expression in (C) 293T and (D) A549 cells. Respective isotype control was used as negative control. (E) Ectopic expression of Sigmodon hispidus (Sh)TfR1 expression in 293T cells. 293T cells were transfected with HA-tagged plasmid for transient ShTfR1 expression. Receptor expression was monitored with specific anti HA monoclonal antibody at the same time than infections were carried out. (TIF) [file pntd.0009004.s005.tif]

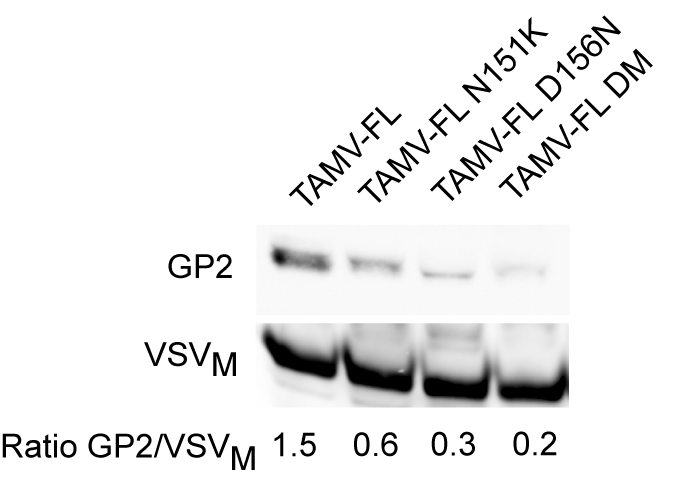

Supplement: S4 Fig — TAMV-FL and TAMV-FL mutant PVs were purified by ultracentrifugation in 30% sucrose cushion (3h, 100000 x g, 4°C), lysed, separated by SDS-PAGE and assayed for TAMV-FL GP2 detection (anti-HA monoclonal antibody) and VSVM (specific VSV-M antibody) in immunoblotting. (TIF) [file pntd.0009004.s006.tif]

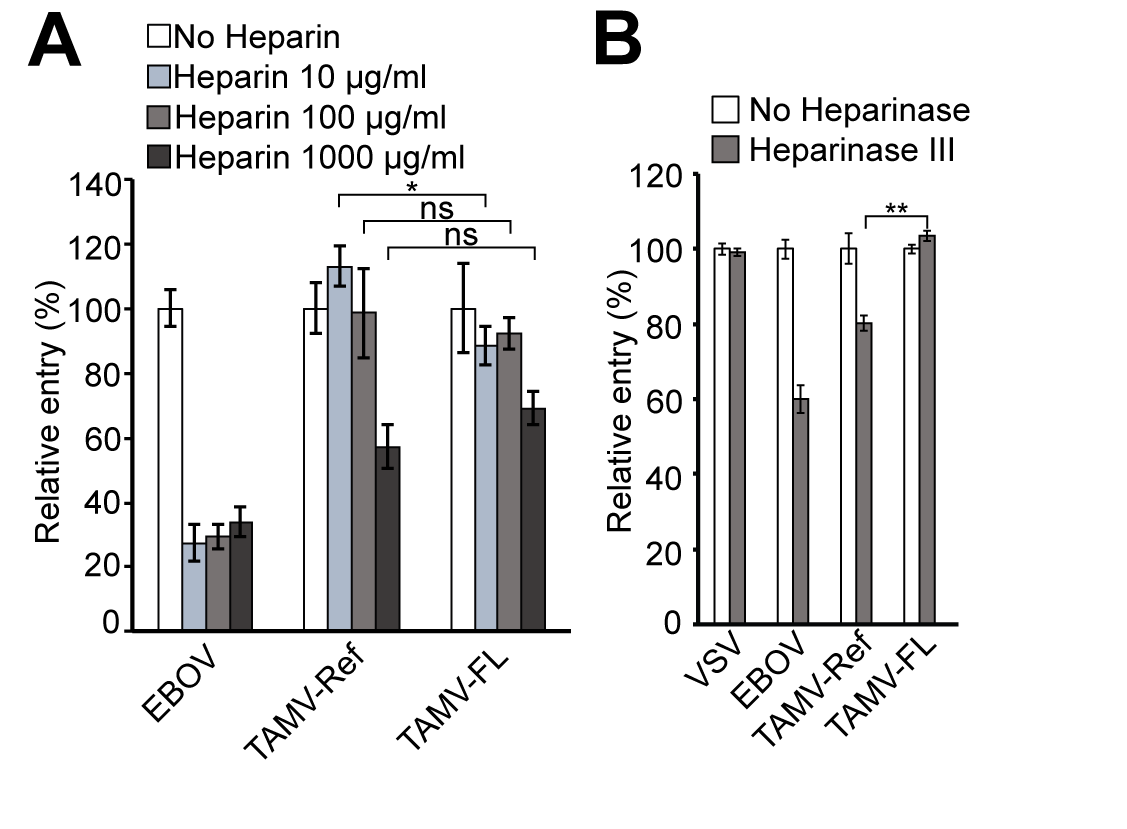

Supplement: S5 Fig — (A) Relative entry in infections performed in A549 cells in presence of increased concentrations of heparin. Error bars represent standard deviations (n = 4). (B) Infections performed in heparinase III-A549-treated cells. Error bars represent standard deviations (n = 4). Asterisks in all panels denote statistical significance in ANOVA test (ns: p>0.05; *: p<0.05; **: p<0.01). (TIF) [file pntd.0009004.s007.tif]

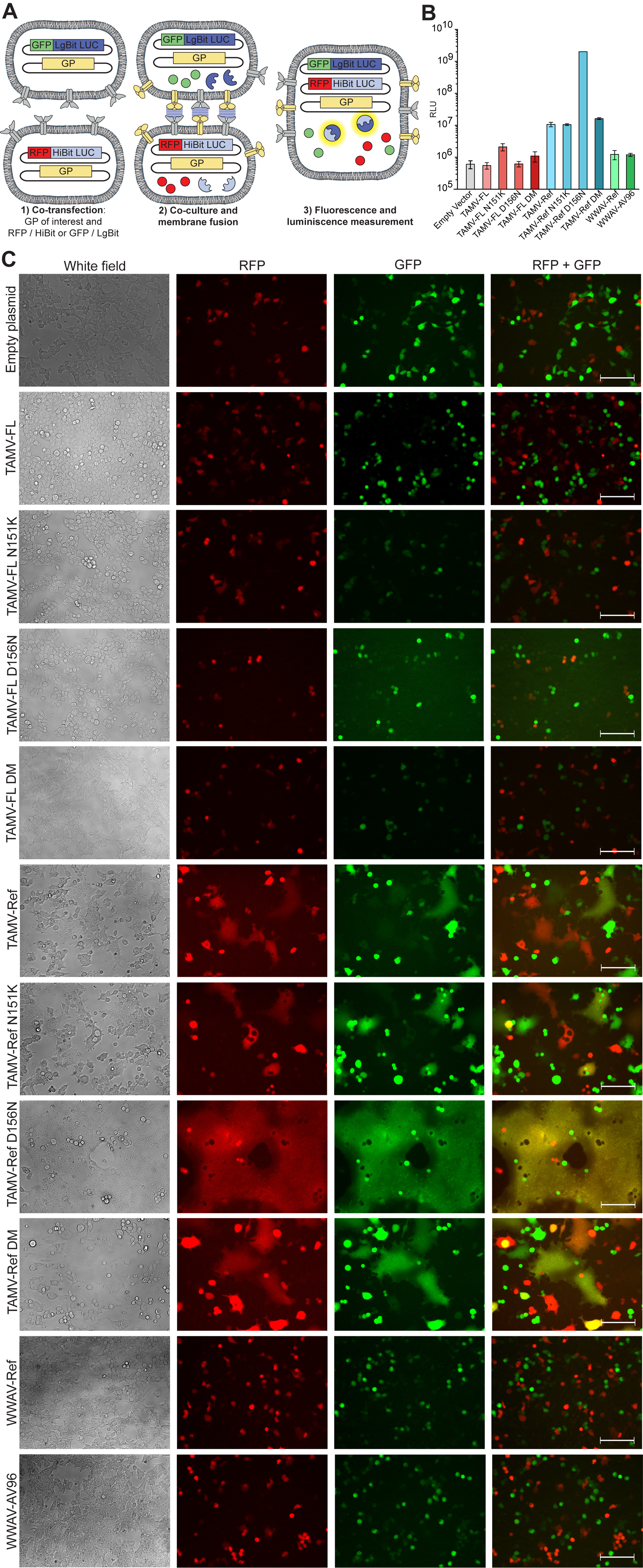

Supplement: S6 Fig — (A) Scheme of syncytia formation monitoring by co-culture of co-transfected 293T cells. LgBit LUC and HiBit LUC protein fragments are only functional when co-expressed in the same cell. Syncytia formation upon clade D NW arenavirus GP transfection in 293T cells monitored by (B) luciferase activity (error bars represent standard deviations of n = 4) or (C) under fluorescence microscope (Scale bars represent 100 μm). (TIF) [file pntd.0009004.s008.tif]
