# Supplementary material for: A novel circulating tamiami mammarenavirus shows potential for zoonotic spillover
Source: PLoS Negl Trop Dis. 2020 Dec 28;14(12):e0009004. doi: 10.1371/journal.pntd.0009004 (PMC7794035; doi:10.1371/journal.pntd.0009004)
Supplement: S1 Table — (DOCX) [file pntd.0009004.s009.docx]

**TABLE S1:** **Taxonomical reads classification obtained from tick-derived sample (done with Kraken) in the NGS run.**

| **Domain** | **Taxonomy*** | **Reads** |
| --- | --- | --- |
| Viruses | ssRNA viruses; Unassigned; Arenaviridae; Mammarenavirus; others | 6 |
| Viruses | ssRNA viruses; Unassigned; Arenaviridae; Mammarenavirus; Tacaribe mammarenavirus | 99158 |
| Viruses | ssRNA viruses; Unassigned; Arenaviridae; Mammarenavirus; Tamiami mammarenavirus | 621 |
| Viruses | ssRNA viruses; Unassigned; Arenaviridae; Mammarenavirus; Latino mammarenavirus | 4 |
| Viruses | ssRNA viruses; Bunyavirales; Peribunyaviridae; Orthobunyavirus; Shamonda orthobunyavirus | 42 |
| Viruses | dsDNA viruses, no RNA stage; Unassigned; Poxviridae; Unassigned; BeAn 58058 virus | 3 |
| Viruses | dsDNA viruses, no RNA stage; Herpesvirales; Alloherpesviridae; Cyprinivirus; Anguillid herpesvirus 1 | 1 |
| Viruses | RT; Ortervirales; Retroviridae; Gammaretrovirus; Baboon endogenous virus | 2 |
| Viruses | RT; Ortervirales; Retroviridae; Alpharetrovirus; Y73 sarcoma virus | 1 |
| Bacteria | Proteobacteria | 4996 |
| Bacteria | Firmicutes | 753 |
| Bacteria | Cyanobacteria | 20 |
| Bacteria | Actinobacteria | 8 |
| Bacteria | Bacteroidetes | 5 |
| Bacteria | others | 159 |
| Unclassified | others | 1 |

*Only the Phylum is shown for bacterial assigned reads
