# Supplementary material for: A novel circulating tamiami mammarenavirus shows potential for zoonotic spillover
Source: PLoS Negl Trop Dis. 2020 Dec 28;14(12):e0009004. doi: 10.1371/journal.pntd.0009004 (PMC7794035; doi:10.1371/journal.pntd.0009004)
Supplement: S2 Table — (DOCX) [file pntd.0009004.s010.docx]

**TABLE S2: Taxonomical contig classification obtained from tick-derived sample (done with Blastn) in the NGS run.**

| **Contig** | **Contig length** | **Subject** | **Subject length** | **Identity (%)** | **E-value** |
| --- | --- | --- | --- | --- | --- |
| NODE_87_cov_96.769013 | 7201 | NC_010702.1 Tamiami virus segment L | 7143 | 87.29 | 0 |
| NODE_98_cov_712.896648 | 6929 | MT478050.1 Tacaribe virus segment L | 7102 | 99.87 | 0 |
| NODE_1086_cov_158.077524 | 3584 | NC_010701.1 Tamiami virus segment S | 3526 | 85.42 | 0 |
| NODE_3686_cov_2395.875826 | 2398 | MT478051.1Tacaribe virus segment S, | 3432 | 99.91 | 0 |
| NODE_29501_cov_3.000994 | 1133 | NC_038668.1 harvey murine sarcoma virus | 997 | 93.09 | 0 |
| NODE_4903_cov_2.343689 | 2185 | NC_001501.1 Moloney murine leukemia virus | 8332 | 73.65 | 7x10^-60^ |
| NODE_4903_cov_2.343689 | 2185 | NC_001940.1 Feline leukemia virus | 8448 | 74.77 | 5 x10^-71^ |
| NODE_5753_cov_13.823560 | 2071 | NC_038922.1 Avian sarcoma virus CT10 | 2428 | 80.3 | 6 x10^-125^ |
| NODE_201_cov_5.965949 | 5795 | NC_001501.1 Moloney murine leukemia virus | 8332 | 77.9 | 1 x10^-86^ |
