# Supplementary material for: A novel circulating tamiami mammarenavirus shows potential for zoonotic spillover
Source: PLoS Negl Trop Dis. 2020 Dec 28;14(12):e0009004. doi: 10.1371/journal.pntd.0009004 (PMC7794035; doi:10.1371/journal.pntd.0009004)
Supplement: S3 Table — (DOCX) [file pntd.0009004.s011.docx]

| **S Segment** | | | | |
| --- | --- | --- | --- | --- |
|  | TCRV-BEI | TCRV-Florida | TCRV-11573 | TCRV-FL |
| TCRV-FL | 5 | 33 | 18 | 0 |
| TCRV-11573 (MT081316.1) | 13 | 25 | 0 |  |
| TCRV-Florida (KF923400.1) | 28 | 0 |  |  |
| TCRV-BEI (MT478051.1) | 0 |  |  |  |
|  |  |  |  |  |
| **L Segment** | | | | |
|  | TCRV-BEI | TCRV-Florida | TCRV-11573 | TCRV-FL |
| TCRV-FL | 90 | 84 | 92 | 0 |
| TCRV-11573 (MT081317.1) | 14 | 10 | 0 |  |
| TCRV-Florida (KF923401.1) | 8 | 0 |  |  |
| TCRV-BEI (MT478050.1) | 0 |  |  |  |

**Table S3.Sequence comparison of TCRV-FL, TCRV-11573, TCRV-Florida and TCRV-BEI.** Single Nucleotide Polymorphisms (SNPs) are shown for each TCRV strain.
