# Supplementary material for: A novel circulating tamiami mammarenavirus shows potential for zoonotic spillover
Source: PLoS Negl Trop Dis. 2020 Dec 28;14(12):e0009004. doi: 10.1371/journal.pntd.0009004 (PMC7794035; doi:10.1371/journal.pntd.0009004)
Supplement: Attachment — Submitted filename: Response to Reviwers (PNTD).docx [file pntd.0009004.s012.docx]

**Response to Reviewers**

Dear Sir, dear Madam,

Please, find below the addressed comments from Reviewers from PLoS Pathogens revision process of the manuscript entitled «A NOVEL CIRCULATING TAMIAMI MAMMARENAVIRUS SHOWS POTENTIAL FOR ZOONOTIC SPILLOVER".

In addition to the indicated modifications to specifically address the Reviewer’s comments, we have also included other changes in the manuscript to make a clearer description of our findings. In particular, these changes concern the Discussion section, which, as suggested by the Reviewer #2 (Minor comments), has been restructured for a better narrative.

Sincerely yours,

Héctor Moreno, PhD.

Lausanne, 8.7.2020.

**Part I – Summary:** Please use this section to discuss strengths/weaknesses of study, novelty/significance, general execution and scholarship.

Reviewer #1: The study by Moreno et al. is an important and timely contribution to the fields of arenavirology and emergent viral infections. They report the isolatation of a new TAMV strain from ticks, first by identifying related arenavirus sequences by deep sequencing of cell culture-adapted viruses from tick homogenates, and taking advantage of superinfection exclusion to enrich passaged viral populations in the new TAMV-FL. Amino acid substitutions associated with a shift towards recognition of the human transferrin receptor 1 and other hos-cell interaction features such as fusion and endosomal escape kinetics are reported. Methods and results are described in sufficient detail, and the discussion is focused on the key points. The study documents the potential of ticks as reservoirs of arenaviruses that upon changes which are commensurate with quasispecies dynamics can acquire mutations to become potential human pathogens.

**Response:** We appreciate the overall positive assessment of our work and the constructive comments. Please find below our specific responses to each of the comments.

Some minor suggestions:

1)	Although statistical significances are reported in the text for some comparative measurements, they should be extended to others, particularly the data shown in Figure 2C, 2D.

**Response:** The statistical analyses of all comparisons has been now included in the text (Lines 201, 206-8, 317-8, 808-9 and 853-4) and figures 2 and 5 have been modified to indicate statistical significance.

2)	Line 148: provide numerical reference.

**Response:** “Sayler et al., 2015” is now numerically referenced.

3)	Explain in Methods why 293 and A549 cells were used for the parallel objective of silencing and knockdown.

**Response:** Lines 721-23 and 739-41 now include a brief description of the reasons why we used A549 cells for CRISPR/Cas9 and 293T for siRNA transient reduced expression of hTfR1.

4)	Line 69: “represents”.

**Response:** The misspelling has been corrected.

5)	S and L for short and long passage do not seem the best abbreviations for an arenavirus manuscript, perhaps regular (not capital) s and l could be used.

**Response:** Fig 3C and D have been modified and “s” and “l” (lower caps), are now used to designate short and long format passages, respectively. The modification has been also applied in lines 253, 255, 259, 263, 265, 271, 273, 281 (title), 505, 510 and 820-1. Table 1 and Figure 5 were also modified accordingly.

6)	Increase of affinity for heparan sulfate upon virus adaptation to cell culture was first described for foot-and-mouth disease virus (T. Jackson et al. J. Virol. 70: 5282, 1996).

**Response:** As suggested by the reviewer, an introductory sentence with suggested reference was added in Results’ section (line 333-4) and cited in lines 541-2.

**Reviewer #2:** This paper by Moreno and colleagues describes the identification of a novel strain of the New World (NW) mammarenavirus Tamiami virus (TAMV), referred to as TAMV-Florida (TAMV-FL), in the host-seeking tick Amblyoma americanum. The authors have provided evidence supporting that TAMV-FL appears to rapidly adapt to grow in mammalian cells via mutations in the virus surface glycoprotein complex that enhance virus dependence on the human transferrin receptor 1 (hTfR1) as well as changes on the optimal pH for the GP2-mediated fusion event between viral and cellular membranes within the endosome compartment, a step that is required to complete the entry process.

The identification of TAMV-FL in ticks further contributes to existing evidence supporting that arthropods may serve as hosts for some mammarenaviruses, which has potential important implications for understanding the epidemiology and zoonotic potential of mammarenaviruses.

The experimental procedures used for the identification of TAMV-FL, and the characterization of mutations within the virus GP contributing to virus adaptation to human cells, are sound and for the most the experiments were carefully conducted including all the appropriate controls.

The main limitation and weakness of the paper is the lack of studies using an isolate of TAMV-FL. The identification of TAMV-FL was based on sound and convincing NGS data. Likewise, the authors designed an elegant and clever strategy to overcome the problems posed by the presence of Tacaribe virus (TCRV), another NW mammarenavirus, in the same Amblyoma americanum biological samples used to screen for novel mammarnaviruses that resulted in the identification of TAMV-FL. However, the authors did not proceed with the isolation and basic characterization of TAMV-FL, but rather for their studies they used the sequence information to generate VSV pseudotyped with TAMV-FL GP. This is certainly a valid approach, but considering that they are describing a novel TAMV isolate, it would be important to complete a basic characterization of TAMV-FL.

This work does not document a novel concept, as mammarenavirus zoonotic events have been documented, as well as factors contributing to an efficient zoonotic event. However, the paper provide interesting details about how specific mutations can be rapidly selected and facilitate virus propagation in mammalian cells. The issue the authors did not discuss is the possibility that TAMV-FL is already circulating in both arthropods and mammals and the authors are reporting a combination of mutations, among many other possibles, favoring multiplication in human cells.

**Response:** We acknowledge and welcome the comments and critical evaluation of the Reviewer. Please, find below our responses to each particular comment.

**Reviewer #3:** In this manuscript by Moreno et al. the authors identify a novel strain of Tamiami virus from ticks and investigate the the receptor usage as a readout for zoonotic potential. The scope of the manuscript is interesting, the work is novel, and the work in itself presented here is sound in the way it was conducted. However, the experimental design and the data presented here does not address the question of zoonotic spillover comprehensively and the statements made in the manuscript are far overreaching.

**Response:** We appreciate critical comments and questions posed by the Reviewer. Please, find below our answers to each particular comment.

--------------------

**Part II – Major Issues:** Key Experiments Required for Acceptance. Please use this section to detail the key new experiments or modifications of existing experiments that should be “absolutely” required to validate study conclusions. Generally, there should be no more than 3 such required experiments or major modifications for a "Major Revision" recommendation. If more than 3 experiments are necessary to validate the study conclusions, then you are encouraged to recommend "Reject".

**Reviewer #1:** None

**Reviewer #2:** The experimental section has been for the most well executed but in many cases the differences shown are rather modest and even if proven to be statistically significant, their biological relevance remains unclear. This concerns applies to Fig 2B, 2C and 2D; some of the results presented in Fig 5 and Fig 6.

Some of these issues are likely to be clarified by the incorporation of experiments using TAMV-FL rather than VSV pseudo types.

The basic characterization of TAMV-FL multiplication in cultured cells, ideally both arthropod and mammalian systems, is important as the virus is being described for the first time.

**Response:** We agree with reviewer #2 that characterization of a unmodified isolate of TAMV-FL would be very interesting, since the biological consequences of the observed genotypic and phenotypic changes in the TAMV-FL GP is one of our current interests. However, we think that this subject goes beyond the scope of the present study, which focuses on the adaptive steps towards a more efficient utilization of hTfR1, which is a critical mammarenavirus trait to evaluate potential human adaptation. In the present study, we benefited from the superinfection exclusion ability of TAMV to overcome TCRV from the original tick-derived sample. Unfortunately, during the passages, TAMV-FL changed its quasispecie composition, in particular, acquired, and enriched the mutations N151K and D156N within the viral population. Therefore, given that TAMV-FL only exists in mixture with TCRV in the original isolate or together with TAMV-FL N151K and/or D156N mutants in the TAMV-FLp5 (either short or long formats), obtaining a purified TAMV-FL, as it existed in the original isolate, is challenging. A way to possibly isolate a purified TAMV-FL without major changes in quasispecies complexity could be doing passaging experiments in non-human permissive cells, in the absence of immune selective pressure, such as in VeroE6 or BHK cells. The obtained TAMV-FL population should be subsequently analyzed by NGS, in order to verify the identity and mutational spectrum of the obtained virus. The selection, population sequencing analyses and the characterization of an unmodified TAMV-FL isolate constitutes a significant amount of work that deserves a separated study, currently under development.

However, to acknowledge potential limitations of the present study, we commented this issue in lines 550-55 and 559-62.

**Reviewer #3:** I believe that there are major components missing in order to assess the spillover potential of TAMV-FL, and the study presented here does not add enough to make these statements.

Major issues:

-The virus was sequenced out of tick pools. This fact gives no proof if the transmission from ticks to humans actually occurs. Isolation of the virus from questing adult ticks -specifically salivary glands- would have been ideal and could have given proof. Maybe this was addressed in other publications, but these references are not listed or discussed. Nowadays, many viruses are found in ticks by NGS, however, this can always be an artifact and gives no evidence if the viruses can be infect transmitted.

**Response:** Although we agree with reviewer #3 that describing not a potential but an actual spillover of TAMV-FL would be certainly interesting, the present study does not aim at demonstrating transmission of TAMV-FL from ticks to humans, and therefore the presented data do not prove it. Instead, our work focuses on the evolutionary steps towards efficient replication in mammalian cells of a virus already circulating among arthropods. In that context, the finding of TAMV in ticks is important because it provides a deeper understanding of mammarenavirus biology. Moreover, it constitutes indeed proof of the fact that circulating TAMV-FL have potential for zoonotic spillover to humans, regardless of whether the transmission occurs from rodents or from arthropods.

Proving transmission from ticks to humans would involve a completely different set of experiments (including, for instance, isolating the virus from clinical samples of human individuals).

To clarify this, we included comments in lines 177-80, 550-55 and 559-62 of the text.

- Why were A549, BHKs or Vero cells chosen? If the virus is supposedly transmitted by ticks, one would look for initial replication in cutaneous tissues where the first adaptation of the virus to the new host i.e. spillover actually occurs.

**Response:** BHK nor VeroE6 cells do not have an operational IFN-I system (1, 2) and both are susceptible to mammarenavirus infection (3-10). Given these characteristics, BHK and VeroE6 cells are typically used to expand viral populations and to perform viral titrations (9, 11-14), respectively. Zoonotic transmission of mammarenaviruses occurs mostly by inhalation of aerosolized contaminated materials (15-18) and therefore the pulmonary epithelial cells A549 permits recapitulating the natural infection process (3, 5-8). Importantly, A549 cells are immunocompetent and susceptible to infection by mammarenaviruses (3, 5, 6, 8-10, 19, 20). Because of this, A549 cells are the gold standard for infection by mammarenaviruses(5-8). To clarify this point, we introduced appropriated comments in lines 44, 86, 122, 193-5, 464-5, and added the references to the text.

-Why were only cell lines and not primary human cells chosen or lat least used to back up. The cell lines used here are known to be IFN-deficient, which introduces a bias to the selection process. Findings should have been backed up on in interferon-competent cells or preferably primary cells.

**Response:** We disagree with reviewer #3. Although BHK and Vero6 cells are immunodeficient, A549 are highly responsive to IFN treatment and trigger a robust IFN-I response upon mammarenavirus infection (3, 5, 6, 8-10, 19, 20). As commented in the text (lines 219-24), for the passages of the tick-derived isolate, we used A549 cells and IFN pre-treatment (100 IU/ml), which indeed trigger a robust and complete IFN-I response (9). That allowed us to introduce a strong selective pressure derived from the activated innate immune response during the passages.

-The main flaw of the study is that the passaging mof the virus was not conducted under any kind of selection/immune pressure rendering the adaptation mutation basically meaningless in regards to adaption under real conditions.

**Response:** In order to demonstrate if TAMV was able to overcome TCRV presence and therefore obtain a purified TAMV population, we performed serial passages in A549 cells. As discussed above, A549 have a functional IFN-I system and respond to IFN-I treatment and mammarenavirus infection (3, 5, 6, 8-10, 19, 20). Moreover, to further enhance this response, and therefore exert a more restrictive selective pressure, we pre-treated A549 cells with IFN (100 IFN/ml), mimicking infection in a cellular environment already induced to an antiviral state.

The enriching mutations N151K and D156N in GP might be just an artifact of the system (no IFN system, no immune pressure) and ultimately is just an easy adaption to the provided environment. How much can really be concluded about spillover from the data?

**Response:** As stated in the two previous points, the passages were performed under conditions of strong immune selective pressure (IFN-stimulated A549 cells), refuting the occurrence of artifacts due to the lack of it.

A comprehensive assessment of the viral capacity for spillover would require a catalogue of experimental approaches, from epidemiology to *in vitro* and *in vivo* assays, in order to completely characterize the plethora of genotypes and phenotypes potentially involved in the infection process. However, the ability to use hTfR1 is a critical factor during adaptation towards human infection (21-25). The present study demonstrates that TAMV-FL can use hTfR1 to enter target cells, which reveals the potential of TAMV-FL for infecting human tissues, and therefore for zoonotic spillover. Although we agree with reviewer #3 that the study of additional factors required or involved in TAMV-FL infection would be required in order to demonstrate the **effective** transmission of TAMV-FL to humans, we think our findings constitute enough evidence for the **potential** of TAMV-FL for zoonotic transmission.

However, to highlight this in the manuscript, and to address a previous point of the Reviewer, we included a sentence in lines 177-80, 550-55 and 559-62.

--------------------

**Part III – Minor Issues:** Editorial and Data Presentation Modifications. Please use this section for editorial suggestions as well as relatively minor modifications of existing data that would enhance clarity.

**Reviewer #1:** Listed in Part I

**Reviewer #2:** The discussion section of the paper could be shortened without having an impact on its scientific content, as in its present form contains some repetition with the information already presented in the results section.

**Response:** Following Reviewer’s comment, we have rearranged the Discussion section to minimize redundancies between sections in the manuscript. To allow highlighting of previous modifications, this modification was not highlighted, although it can be viewed by tracked-modification system in Microsoft Word.

**Reviewer #3:** none

--------------------

PLOS authors have the option to publish the peer review history of their article ("<https://journals.plos.org/plospathogens/s/editorial-and-peer-review-process#loc-peer-review-history>" what does this mean? If published, this will include your full peer review and any attached files.)

If you choose “no”, your identity will remain anonymous but your review may still be made public.

Do you want your identity to be public for this peer review?For information about this choice, including consent withdrawal, please see "<https://www.plos.org/privacy-policy>".

Reviewer #1: No

Reviewer #2: No

Reviewer #3: No

__________________________________________________

In compliance with data protection regulations, you may request that we remove your personal registration details at any time. (Use the following URL: <https://www.editorialmanager.com/ppathogens/login.asp?a=r>). Please contact the publication office if you have any questions.

**REFERENCES :**

1.	Stanwick TL, Hallum JV. Role of interferon in six cell lines persistently infected with rubella virus. Infect Immun. 1974;10(4):810-5.

2.	Andzhaparidze OG, Bogomolova NN, Boriskin YS, Bektemirova MS, Drynov ID. Comparative study of rabies virus persistence in human and hamster cell lines. J Virol. 1981;37(1):1-6.

3.	Huang C, Kolokoltsova OA, Yun NE, Seregin AV, Poussard AL, Walker AG, et al. Junin virus infection activates the type I interferon pathway in a RIG-I-dependent manner. PLoS Negl Trop Dis. 2012;6(5):e1659.

4.	Huang C, Kolokoltsova OA, Yun NE, Seregin AV, Ronca S, Koma T, et al. Highly Pathogenic New World and Old World Human Arenaviruses Induce Distinct Interferon Responses in Human Cells. J Virol. 2015;89(14):7079-88.

5.	Mateer EJ, Paessler S, Huang C. Visualization of Double-Stranded RNA Colocalizing With Pattern Recognition Receptors in Arenavirus Infected Cells. Front Cell Infect Microbiol. 2018;8:251.

6.	Mateer EJ, Maruyama J, Card GE, Paessler S, Huang C. Lassa Virus, but Not Highly Pathogenic New World Arenaviruses, Restricts Immunostimulatory Double-Stranded RNA Accumulation during Infection. J Virol. 2020;94(9).

7.	Martinez-Sobrido L, Giannakas P, Cubitt B, Garcia-Sastre A, de la Torre JC. Differential inhibition of type I interferon induction by arenavirus nucleoproteins. J Virol. 2007;81(22):12696-703.

8.	Rodrigo WW, Ortiz-Riano E, Pythoud C, Kunz S, de la Torre JC, Martinez-Sobrido L. Arenavirus nucleoproteins prevent activation of nuclear factor kappa B. J Virol. 2012;86(15):8185-97.

9.	Moreno H, Moller R, Fedeli C, Gerold G, Kunz S. Comparison of the Innate Immune Responses to Pathogenic and Nonpathogenic Clade B New World Arenaviruses. J Virol. 2019;93(19).

10.	King BR, Hershkowitz D, Eisenhauer PL, Weir ME, Ziegler CM, Russo J, et al. A Map of the Arenavirus Nucleoprotein-Host Protein Interactome Reveals that Junin Virus Selectively Impairs the Antiviral Activity of Double-Stranded RNA-Activated Protein Kinase (PKR). J Virol. 2017;91(15).

11.	Grande-Perez A, Sierra S, Castro MG, Domingo E, Lowenstein PR. Molecular indetermination in the transition to error catastrophe: systematic elimination of lymphocytic choriomeningitis virus through mutagenesis does not correlate linearly with large increases in mutant spectrum complexity. Proc Natl Acad Sci U S A. 2002;99(20):12938-43.

12.	Battegay M, Cooper S, Althage A, Banziger J, Hengartner H, Zinkernagel RM. Quantification of lymphocytic choriomeningitis virus with an immunological focus assay in 24- or 96-well plates. J Virol Methods. 1991;33(1-2):191-8.

13.	Rojek JM, Perez M, Kunz S. Cellular entry of lymphocytic choriomeningitis virus. J Virol. 2008;82(3):1505-17.

14.	Borrow P, Oldstone MB. Mechanism of lymphocytic choriomeningitis virus entry into cells. Virology. 1994;198(1):1-9.

15.	Stephenson EH, Larson EW, Dominik JW. Effect of Environmental-Factors on Aerosol-Induced Lassa Virus-Infection. Journal of Medical Virology. 1984;14(4):295-303.

16.	terMeulen J, Lukashevich I, Sidibe K, Inapogui A, Marx M, Dorlemann A, et al. Hunting of peridomestic rodents and consumption of their meat as possible risk factors for rodent-to-human transmission of Lassa virus in the Republic of Guinea. American Journal of Tropical Medicine and Hygiene. 1996;55(6):661-6.

17.	Rodas JD, Lukashevich IS, Zapata JC, Cairo C, Tikhonov I, Djavani M, et al. Mucosal Arenavirus infection of primates can protect them from lethal hemorrhagic fever. Journal of Medical Virology. 2004;72(3):424-35.

18.	Peters CJ. Human infection with arenaviruses in the Americas. Curr Top Microbiol. 2002;262:65-74.

19.	Huang C, Kolokoltsova OA, Mateer EJ, Koma T, Paessler S. Highly Pathogenic New World Arenavirus Infection Activates the Pattern Recognition Receptor Protein Kinase R without Attenuating Virus Replication in Human Cells. J Virol. 2017;91(20).

20.	Pythoud C, Rothenberger S, Martinez-Sobrido L, de la Torre JC, Kunz S. Lymphocytic Choriomeningitis Virus Differentially Affects the Virus-Induced Type I Interferon Response and Mitochondrial Apoptosis Mediated by RIG-I/MAVS. J Virol. 2015;89(12):6240-50.

21.	Radoshitzky SR, Abraham J, Spiropoulou CF, Kuhn JH, Nguyen D, Li W, et al. Transferrin receptor 1 is a cellular receptor for New World haemorrhagic fever arenaviruses. Nature. 2007;446(7131):92-6.

22.	Abraham J, Kwong JA, Albarino CG, Lu JG, Radoshitzky SR, Salazar-Bravo J, et al. Host-species transferrin receptor 1 orthologs are cellular receptors for nonpathogenic new world clade B arenaviruses. Plos Pathog. 2009;5(4):e1000358.

23.	Flanagan ML, Oldenburg J, Reignier T, Holt N, Hamilton GA, Martin VK, et al. New world clade B arenaviruses can use transferrin receptor 1 (TfR1)-dependent and -independent entry pathways, and glycoproteins from human pathogenic strains are associated with the use of TfR1. J Virol. 2008;82(2):938-48.

24.	Reignier T, Oldenburg J, Flanagan ML, Hamilton GA, Martin VK, Cannon PM. Receptor use by the Whitewater Arroyo virus glycoprotein. Virology. 2008;371(2):439-46.

25.	Shimon A, Shani O, Diskin R. Structural Basis for Receptor Selectivity by the Whitewater Arroyo Mammarenavirus. J Mol Biol. 2017;429(18):2825-39.
