# Supplementary material for: A novel circulating tamiami mammarenavirus shows potential for zoonotic spillover
Source: PLoS Negl Trop Dis. 2020 Dec 28;14(12):e0009004. doi: 10.1371/journal.pntd.0009004 (PMC7794035; doi:10.1371/journal.pntd.0009004)
Supplement: Attachment — Submitted filename: Response to reviewers R2 PNTD.docx [file pntd.0009004.s013.docx]

Dear Sir, dear Madam,

Please, find below the addressed comments and questions from the revision process of the manuscript entitled «A NOVEL CIRCULATING TAMIAMI MAMMARENAVIRUS SHOWS POTENTIAL FOR ZOONOTIC SPILLOVER".

We appreciate very much the scientifically enriching, critical, and constructive comments from all Reviewers. We have addressed all comments, one by one, highlighting our responses in blue.

Sincerely yours,

Héctor Moreno, PhD.

Lausanne, 31.7.2020.

Dear Dr. Moreno,

Thank you very much for submitting your manuscript "A NOVEL CIRCULATING TAMIAMI MAMMARENAVIRUS SHOWS POTENTIAL FOR ZOONOTIC SPILLOVER" for consideration at PLOS Neglected Tropical Diseases. As with all papers reviewed by the journal, your manuscript was reviewed by members of the editorial board and by several independent reviewers. In light of the reviews (below this email), we would like to invite the resubmission of a significantly-revised version that takes into account the reviewers' comments.

All three reviewers express interest in the study, but one reviewer have several criticisms and suggestions, which need to be addressed.

We cannot make any decision about publication until we have seen the revised manuscript and your response to the reviewers' comments. Your revised manuscript is also likely to be sent to reviewers for further evaluation.

When you are ready to resubmit, please upload the following:

[1] A letter containing a detailed list of your responses to the review comments and a description of the changes you have made in the manuscript. Please note while forming your response, if your article is accepted, you may have the opportunity to make the peer review history publicly available. The record will include editor decision letters (with reviews) and your responses to reviewer comments. If eligible, we will contact you to opt in or out.

[2] Two versions of the revised manuscript: one with either highlights or tracked changes denoting where the text has been changed; the other a clean version (uploaded as the manuscript file).

Important additional instructions are given below your reviewer comments.

Please prepare and submit your revised manuscript within 60 days. If you anticipate any delay, please let us know the expected resubmission date by replying to this email. Please note that revised manuscripts received after the 60-day due date may require evaluation and peer review similar to newly submitted manuscripts.

Thank you again for your submission. We hope that our editorial process has been constructive so far, and we welcome your feedback at any time. Please don't hesitate to contact us if you have any questions or comments.

Sincerely,

Jonas Klingström

Associate Editor

PLOS Neglected Tropical Diseases

Jeremy Camp

Deputy Editor

PLOS Neglected Tropical Diseases

***********************

**Reviewer's Responses to Questions**

<b>Key Review Criteria Required for Acceptance?</b></br></br> As you describe the new analyses required for acceptance, please consider the following:</br></br>

<b>Methods</b></br></br>

-Are the objectives of the study clearly articulated with a clear testable hypothesis stated?</br> -Is the study design appropriate to address the stated objectives?</br> -Is the population clearly described and appropriate for the hypothesis being tested?</br> -Is the sample size sufficient to ensure adequate power to address the hypothesis being tested?</br> -Were correct statistical analysis used to support conclusions?</br> -Are there concerns about ethical or regulatory requirements being met?</br></br>

**Reviewer #1:** The methods used are appropriate, as well as the data analysis.

We appreciate the Reviewer’s comment on the methods and experimental set up in our study.

**Reviewer #2:** 1) Despite the request of the previous reviewers, statistical assessment is still missing for some experiments (e.g. Figs 2E, 6 and 7, S2 and S3B). Further, many of the figures fails to indicate what statistical test was performed and this information also appears to be missing in the methods. Where the test is indicated, the authors say they used a T-test, which is inappropriate for the analysis of datasets containing more than 2 samples (even where only pair-wise comparisons of samples are subsequently analysed). Rather ANOVA needs to used.

As suggested by the Reviewer, we have now included statistical analyses in the figures 2E, 6, S2 and S3B. As the statistical analysis of half-escape time and IC50 values were denoted in Tables 2-4, we did not represent it on Figure 7. Furthermore, as suggested by the Reviewer, we have re-analyzed the dataset using ANOVA tests and included a description of the method used in the Material and Methods sections (lines 849-52).

2) Lines 206-208. Here (and also elsewhere in the text) the authors appear to be trying to use the strength of their p values as an indication of the strength of their biological effect. This is not what p-values indicate and they simply cannot be used in this way.

We completely agree with the Reviewer, and p values cannot be interpreted as an indicator of the biological effect of an observation. Therefore, to avoid possible misinterpretations of the p values, we have modified the following lines: 227, 234, 236, 241, 242 and 257-8.

3) In assessing TAMV strain diversity as it relates to their new TAMV-FL isolate the authors need to include values for the comparison to the much more recently isolated TAMV sequence for strain CDC W-10777 from 1997, which is publically available (e.g. In GenBank).

We acknowledge the comment from the Reviewer regarding a faithful comparison of TAMV available complete sequences. Although isolated in 1960s, TAMV W-10777 (TAMV-Ref in our study) was not completely sequenced until 2002 (1). In 2008 (2), a new TAMV W-10777 sequence was added, showing only 11 SNV in difference with the previously sequenced TAMV W-10777. Since then, no additional TAMV complete sequences have been added to GenBank.

In agreement with the Reviewer’s comment, we have now included a table (Table 1) and a supplementary figure (Fig. S1) showing the homology and alignments, respectively, with aforementioned available TAMV sequences. Moreover, we have commented this in lines 495-8.

4) The authors need to clearly indicate the provenance of their sample in the methods. This is vaguely alluded to in the acknowledgements and the use of references, but it needs to be made clear if this is the exact same tick pool that Sayler et al. used in their publication of a newly identified TCRV sequence. If this is the case, it is not surprising that the authors observe a TCRV sequence with similarly high sequence identity to that previously reported, whereas if this is a different tick pool this observation gains increased significance.

In either case, it is important to note that the choice of reference sequence used by the authors for their analysis of the TCRV sequence is not suitable, as this sequence has been repeatedly shown to contain major discrepancies compared to all other sequences of the same isolate (e.g. PMID: 26587982 (accession: KP159416 ), PMID: 23382389 (accession: KC329849)) and that these account for almost all of the difference between the Florida and the original TRVL-11573 isolates (PMID: 32462284, accession numbers: MT081316, MT081317). Any analysis of their identified TCRV sequences should use these newer sequences for comparison.

In agreement with the Reviewer’s comment, we have clarified the tick-derived isolate origin in lines 154-5 and 655-8. Moreover, following the Reviewer’s comment, we have re-analyzed our TCRV-FL sequences and compared with the most recent TCRV sequences (MT081316.1, MT081317.1, KF923400.1, KF923401.1, MT478051.1 and MT478050.1) (3-5). Results are now shown in Table S3 and commented in lines 165-76. As indicated by the Reviewer, we have corrected Figure 1 and table S2, which now show the results of comparison between TCRV-FL with TCRV-Bei strain (MT478051.1 and MT478050.1 (4).

**Reviewer #3:** The methods are appropriate. Experiments have been conducted according to high standards and have been controlled via appropriate controls.

We thank the Reviewer’s comment and evaluation of our experiments and results.

--------------------

<b>Results</b></br></br>

-Does the analysis presented match the analysis plan?</br> -Are the results clearly and completely presented?</br> -Are the figures (Tables, Images) of sufficient quality for clarity?</br></br>

**Reviewer #1:** The results are adequately presented.

We appreciate the evaluation from the Reviewer of the results presented in our study.

**Reviewer #2:** 1) I can appreciate that the requests of the previous reviewers to obtain a non-adapted TAMV-FL for confirmatory work to support the authors’ findings with VSV pseudotypes, may go beyond the scope of the present study. However, if the authors are going to depend exclusively on these pseudotype data for some of their work, they need at least to confirm that their VSV pseudotypes contain equivalent amounts of GP incorporation. Indeed, could such differences possibly explain for example the striking difference in the role of hTfR1 in WWAV when using antibody vs KD cells (Fig 5A vs 5B)? This leads the authors to strongly assert that WWAV-Ref does not use hTfR1 (Line 455-458), although this is actually contradicted by some of their own data (Figure 5B).

Further, if the authors are unable to do confirmatory work with actual virus (which is justifiably the case here) they still need to directly acknowledge this limitation of the study.

We agree with the Reviewer and performed additional experimentation to determine the amount of GP in our PV preparations as well as the relevance of this aspect in the PV infectivity. In first term, we quantified the amounts of GP and VSV-M proteins by immunoblotting in TAMV-Ref, TAMV-FL and TAMV-FL mutants PV preparations (Fig. S2A and S4), and found that, GP was more abundant in TAMV-Ref PV than in TAMV-FL PV particles, as well as that, regardless of the TAMV genetic backbone (FL or Ref), the introduction of the substitutions N151K, D156N, or both (DM), led to a reduction of the amount of GP in the PV preparations. Thus, the increased hTfR1 usage observed in PV carrying the N151K or D156N substitutions is not due to higher amounts of GP in their PV. Indeed, these results suggest that the hTfR1 dependence of the PV harboring N151K or D156N GP mutations might be even underestimated.

We went further and investigated the effect of variations in the amount of GP on viral infectivity of PV, in order to determine if the observed increase of hTfR1 usage could be caused by different amounts of GP in PV preparations. We produced PV with increasing amounts of DNA, and generated PV preparations containing ranged amounts of GP (Fig. S2B). Infections with equal volumes of PV preparations (Fig. S2C) or with volumes normalized with the amount of measured GP (Fig. S2D) indicate that the amount of GP in PV preparations affects PV titer but not their infectivity. In sum, these results demonstrate that the observed differences in the infectivity of the variants carrying N151K and D156N substitutions are not due to differences in GP amounts, and further validate the use of VSV-based pseudoviruses to compare GP-mediated infectivity. To summarize these observations, lines 209-18 and 353-5 were modified. Moreover, in agreement with the Reviewer’s comment, lines 509-11 were modified to comment on the hTfR1 usage of WWAV-AV96.

2) Based on what the authors write in the methods it appears that they did not synchronize their infections for the ammonium chloride escape assays. This is clearly needed in this case since the authors show there are indeed differences in binding between these pseudotypes. Otherwise, the influence on binding may convolute the authors findings, which involve changes in escape time within a very short window. Indeed, might this help explain some of the internal inconsistencies observed in the authors data between the ammonuium chloride concentration and time of escape data? e.g. TAMV-REF and JUNV have the same IC50 but very different escape times, while JUNV and TAMV-FL have the same escape times, but quite different IC50s. Similarly, WWAV-REF and WWAV-AV96, differ substantially in IC50 but have almost identical escape times. The authors also need to clearly address/explain these discrepancies, as they otherwise might call into question the reliability of this assay and/or the biological relevance of differences seen on this time scale.

We agree with the Reviewer regarding the requirement of synchronizing the infections in endosomal escape experiments. We regularly perform this type of experiments in our lab, and synchronizing the infections is the general practice (6). By mistake, we did not include a detailed description of the experimental setup, including the synchronization of infections. We apologize for the omission, that has been corrected this in the revised version of the manuscript by modifying the lines 779-82. Furthermore, we also apologize for a calculation mistake in the ammonium chloride IC_50_ of JUNV in the former table 2 (current Table 3), which is now corrected and correlates with the data in Figure 7.

As stated in the manuscript (Lines 439-41), ammonium chloride sensitivity is an artificial experimental set up that provides information of the fusogenic activity of GPs of interest. Nevertheless, it is conceivable that these results do not completely correlate with endosomal escape kinetics. For instance, participation of additional host factors (such intracellular receptor switch, as observed in the OW mammarenavirus Lassa Virus or Ebola Virus (7-11), can cause apparent discrepancies between both experimental approaches. This is, indeed, a current project in development in our lab. To further clarify this, we have modified lines 457-60.

3) Why do the authors only look at heparin usage but not hTfR1 dependence using their mutants (N151K, D156N and DM) in the TAMV-REF background?

We agree again with the Reviewer and performed additional experimentation to address the influence on hTfR1 usage of N151K, D156N and DM substitutions in the TAMV-Ref backbone. The results show that the introduction of aforementioned substitutions in the TAMV-Ref GP does not increase the hTfR1 binding capacity, as their infectivity are not affected by the presence of an anti-hTfR1 blocking antibody. Lines 365-71 were modified to include this observation, as well as a new panel in figure 5.

**Reviewer #3:** Results are clearly presented in Figures, Tables and text. Figures and Tables are of high quality.

We thank the Reviewer’s comment.

--------------------

<b>Conclusions</b></br></br>

-Are the conclusions supported by the data presented?</br> -Are the limitations of analysis clearly described?</br> -Do the authors discuss how these data can be helpful to advance our understanding of the topic under study?</br> -Is public health relevance addressed?</br></br>

**Reviewer #1:** The conclusions are fully supported by the results.

We thank the Reviewer’s comment and evaluation of our discussion.

**Reviewer #2**: 1) As noted above (and also identified as an issue in the previous revision of the paper for PLoS Pathogens), the authors’ statements appear to misleadingly suggest that the identification of virus in blood-feeding arthropods can be used to presume an epidemiologically relevant function as a vector (e.g. Lines 37-40, Line 110-112, Line 435-439). In the absence of data for efficient virus replication, dissemination with the vector and effective transmission from the vector under relevant biological conditions these statements need to significantly moderated.

Indeed, our findings demonstrate the presence of replicating TAMV in host-seeking ticks, its divergence with previously isolated TAMV strains, and the genetic plasticity shown in cell culture conditions. As commented by the Reviewer, we did not addressed epidemiological aspects such the verification of TAMV replication in ticks or effective vector transmission, and, therefore, we have moderated the statements and conclusions regarding the role of ticks in mammarenavirus epidemiology (Lines 51-2, 126-31, 295-8, and 487-90).

2) Also the authors’ assertion that their TAMV-FL isolate has “evolved to hTfR1 usage” is not necessarily supported by the data. Is it not just as likely (if not more so) that the reference strain of TAMV has evolved to have decreased dependence of hTfR1 as a result of its passage history over the last 50+ years? In that respect the authors also need to provide whatever information they have regarding the passage history of the TAMV isolate from which their TAMV-REF GP sequence is derived.

In agreement with the Reviewer’s comment, and to minimize the possible effect on passage history of the TAMV-Ref strain, we *in vitro* synthetized the GP from the original TAMV-10777 strain sequence, publicly available in GenBank (NC_010701.1 and NC_010702.1) (1). Therefore, in our study we faithfully compared the tick derived TAMV-FL isolate with the original TAMV-Ref, lacking any influence of passage history. To clarify this point we have modified lines 218-21 and 641-3 in the main text.

3) The authors refer to the “striking genetic plasticity” of TAMV (Lines 51-53), however, there is no indication as to what they are comparing to in order to make this assessment? Is the observed degree of variation so unusual for an arenavirus, either with respect to the extent of the divergence seen between the natural TAMV isolates (based on their respective dates of isolation), or in the number of changes seen following their 5 passages when transitioning to a cell culture based on a cell type derived from a different species? A concrete and objective basis for this assessment needs to be provided.

We acknowledge the comment from the Reviewer, and clarified this in lines 51-2 and 495-8. TCRV (strain 11573) and TAMV (strain W-10777) were originally isolated in the 1960s. Nevertheless, the comparison between the changes occurred between TCRV-FL and all available TCRV strains, revealed very limited changes in sequence, suggesting genetic stability over years. On the contrary, TAMV-FL accounted a high number of substitutions, compared to TAMV-Ref (W-10777), which suggests a notable genetic plasticity.

4) Work with the Candid#1 vaccine strain of JUNV shows that it has also acquired increased dependence on hTfR1 compared to virulent JUNV, and here it is posited to be associated with attenuation (PMID: 21976641). Thus it is not clear why the authors expect that in TAMV-FL a similar change due to passaging in human cells would constitute a predilection for adaptation to pathogenicity in humans, as they appear to be suggesting.

The mechanisms underlying the attenuation of JUNV Candid#1 are unclear. As the Reviewer pointed out, contrary to all the pathogenic NW mammarenaviruses, JUNV Candid#1 had higher affinity for the hTfR1 than its pathogenic parental strain (12). Nevertheless, a recent publication (13) identified a single mutation in the JUNV Candid#1 Z protein, which confers attenuation. Therefore, the apparent increase of hTfR1 binding capacity of JUNV Candid#1, and its possible effect on pathogenicity, may be counterbalanced by the mutation in the Z protein, explaining the conflict between increased hTfR1 usage and reduced pathogenicity in humans, and further supporting the hTfR1 as indicator of human pathogenicity (14, 15). To comment on this, lines 511-17 in the main text have been modified.

5) Line 521-524. The authors’ speculation that the observed delay in endosomal escape facilitates immune evasion during transport to the areas in which inclusion bodies occur is highly speculative. Firstly, this model is based on the supposition that genomes have to be transported to the sites of eventual inclusion body formation, which is not necessarily the case as these structures are the product of viral protein accumulation once genome delivery and viral RNA synthesis has already been successfully established. There is no evidence for instance that primary transcription takes place at these sites. Perhaps more importantly though, it overlooks other work on endosomal entry factors for arenaviruses suggesting that they act to enhance infection by facilitating early escape of the virus at higher pH thereby avoiding particle damage at low pH (PMID: 29295909). As such, it is not at all clear that delayed escape would be “a good thing” for the virus. Indeed, its harmful effects appear to be supported to some extent by the authors own observations that their double mutant is strongly disfavoured in nature, suggesting low viability.

As pointed out by the Reviewer, the technical limitations impede to determine the specific location of the early sites of replication of Mammarenaviruses, which is now commented in lines 577-8. In the discussion, we speculated on the possible effect, either positive or negative, on selection for delayed endosomal escape trait. Compared to other mammarenaviruses, LASV GP-mediated fusion occurs at unusually low pH (16, 17). In agreement with this finding, additional studies demonstrated that LAMP1 supports LASV GP mediated membrane fusion at pH ≈5.5 (11), and also that in absence of LAMP1, LASV GP1-mediated syncytia occurs at pH < 4. Moreover, as pointed out by the Reviewer, in (7), the researchers found that in absence of LAMP1, LASV undergoes endosomal escape at less acidic pH, but, importantly, with a high cost in infectivity. In sum, these findings suggest that late endosomal escape might be beneficial for mammarenaviruses, but also that LASV highjacks LAMP1, present in acidic endosomal compartments (pH 4.5-5) (18), maybe to prevent virion disintegration due to excessive acidification. To discuss these aspects, we have modified lines 581-7.

6) How do the authors suggest that the D156N mutant can show enhanced fusion at neutral pH (Figure S3) and yet show, if anything, slightly delayed fusion triggering (i.e. a need for lower pH for fusion) in the ammonium chloride escape assay (Fig 4)?

Our results show that the mutation D156N increases the fusogenic activity only when located in the TAMV-Ref backbone (Figure S5), while it causes a delay in endosomal escape only when located in the TAMV-FL backbone (Figure 7C-D). Therefore, the phenotype of aforementioned mutation depends on the genetic backbone on which is located.

Reviewer #3: All conclusions are balanced and supported by the data. Public health relevance of the data is addressed in the Discussion.

--------------------

<b>Editorial and Data Presentation Modifications?</b></br><br/> Use this section for editorial suggestions as well as relatively minor modifications of existing data that would enhance clarity. If the only modifications needed are minor and/or editorial, you may wish to recommend “Minor Revision” or “Accept”.

**Reviewer #1:** I recommend accepting the manuscript, as is.

We greatly appreciate the Reviewer’s recommendation for the acceptation for publication of our study.

**Reviewer #2:** 1) The authors are encouraged to also submit their TAMV-FL sequences to GenBank and include the corresponding accession numbers in the manuscript text.

We acknowledge the Reviewer’s comment and the sequences corresponding to S and L segments of both, TCRV-FL (Although sequences have been submitted to GenBank, the accession numbers are not still available, and we apologize for the inconvenience.) and TAMV-FL (MK500936 and MK500937) are now uploaded and publicly available in GenBank, as indicated in lines 166 and 176.

2) Line 240-241. The authors should clarify for the reader that passage 2 in the long passage and passage 6 in the short passage amount to the same total amount of time for virus growth – which is also what one might expect.

To reflect the Reviewer’s comment, we have now indicated the total time after infection in line 276.

3) “Frags” and “fragments” in Fig 1E should more correctly be ‘segments”.

As indicated by the Reviewer, the aforementioned figure has been modified. Furthermore, same correction has been done in the main text and figure 5.

4) Lines 442-445 and 459-461 are redundant.

We appreciate the Reviewer’s correction, and we have removed the sentence at lines 517.

5) Lines 469-472 is redundant with respect to the statements about heparin sulfate binding

Sentence in line 526 has been corrected to avoid the redundancy regarding the heparan sulfate binding.

**Reviewer #3:** (No Response)

--------------------

<b>Summary and General Comments</b></br></br> Use this section to provide overall comments, discuss strengths/weaknesses of the study, novelty, significance, general execution and scholarship. You may also include additional comments for the author, including concerns about dual publication, research ethics, or publication ethics. If requesting major revision, please articulate the new experiments that are needed.

**Reviewer #1:** The manuscript by Moreno H. et al. describes the isolation of a new TAMV strain from ticks and the characterization of two amino-acid substitutions that promote hTfR1 and HSPG utilization, affect the spike triggering, as well as the cell penetration dynamics of the virus. The quick adaptation of the virus to hTfR1 suggests a potential capacity for spillover.

This is a highly timely and important manuscript in the arenaviral field. The results are exciting and will certainly contribute to the field.

The manuscript has already gone a thorough round of revision, where the authors addressed comments made by three reviewers.

I found the methods, display of results, and discussion to be appropriate.

Except for a few occasional typos (i.g. line 407 "fig. 9c" should probably be "fig. 7c", for example), the manuscript is of high quality, and I thus recommend accepting it as is.

We thank the Reviewer’s evaluation of our study and his/her recommendation for acceptation for publication. We have also corrected typos, including the one mentioned above, in the manuscript.

**Reviewer #2:** The manuscript by Moreno et al. describes the authors’ NGS sequencing of a new Tamiami virus (TAMV) isolate obtained from tick pools collected in Florida that have been previously reported to contain another arenavirus, Tacaribe virus (TCRV). The TAMV sequence identified is shown to be highly divergent from the reference strain from 1965, and thus may in its own right quite interesting with respect to understanding the extent of genetic variation of this virus. However, to fully appreciate this point the authors need to also include detailed information on comparisons of their sequence to the much more recently isolated TAMV sequences (strain CDC W-10777) from 1997, which are also publically available (e.g. In GenBank).

We appreciate the Reviewer’s comment in our study. Despite being initially isolated in 1960s, TAMV W-10777 was not completely sequenced until 2002 (1). Since then only an additional sequence, also derived from W-10777 strain, was made publicly available (2). As suggested by the Reviewer, to better compare the TAMV-FL sequence with the available sequences, we have now included an alignment of complete available TAMV sequences in Figure S1, Table 1 and commented in lines 481-4.

Based on this sequence the authors go on to generate pseudotyped VSVs containing TAMV-FL, in order to compare usage of the human transferrin receptor and heparin to facilitate psuedotype particle entry. Further, they then use a quite clever approach (based on non-reciprocal superinfection exclusion) to isolate a passaged version of this new TAMV strain. During this process, the authors observe several mutation that become enriched during passage compared to their starting sequence and evaluate the relevance of these mutations for entry, including both receptor usage and the pH dependence of fusion. While these experiments are mostly well done, there a few points of concern that need to be clearly addressed. Specifically, the ammonium chloride escape experiments need to use synchronized infections, and the authors need to confirm that their pseudotypes have comparable (mature) GP incorporation. indeed these points may explain some of issues with internal consistency within the authors data, which they do not address.

As commented above, we apologize for the mistake of not providing a detailed description of how Endosomal escape experiments were performed. In the current manuscript, this has been already corrected in lines 779-82. Moreover, a miscalculation in the ammonium chloride IC_50_ of JUNV (current table 3) has been corrected. Furthermore, the apparent discrepancies between endosomal escape experiments and ammonium chloride sensitivity might suggest participation of additional host factors during viral entry, as already described for the OW mammarenaviruses LASV and Lujo virus, as well as EBOV (7-11, 19), as commented in lines 457-60.

Currently, the major weakness of the paper is that the authors make some broad provocative claims (including in the title) that are based on lines of argumentation that are not well developed and/or outright exceed what can reasonably be concluded based on the data.

We appreciate the critical comment of the Reviewer and we have consequently moderated the interpretations of the study. Among additional modifications along the main text, following the Reviewer’s comments and suggestions, we have:

(I) Clarified the apparent incongruence of the increased hTfR1 dependence of JUNV Candid#1 correlated with a reduced human virulence (Lines 511-16).

(II) Discussed the possible selection towards delayed endosomal escape in mammarenaviruses, and the current limitations in this regard (577-8 and 581-7).

(III) Moderated statements and commented on the biological consequences –and limitations of this study- of the possible participation of ticks in mammarenavirus epidemiology (Lines 51-2, 126-31, 295-8, and 487-90)

**Reviewer #3:** The manuscript presented by Moreno et al. is an important addition to our understanding of adaptation processes that could facilitate an effective spread of arenaviruses from their natural reservoir to humans. The data are original and the experiments were carefully conducted and interpreted. In addition, the authors have responded to all comments of the previous reviewers and have thoroughly revised the manuscript. In my opinion, there is no need for further changes.

We appreciate the Reviewer’s comment and overall evaluation and relevance of our study.

--------------------

PLOS authors have the option to publish the peer review history of their article (<a href="[https://journals.plos.org/plosntds/s/editorial-and-peer-review-process#loc-peer-review-history](https://journals.plos.org/plosntds/s/editorial-and-peer-review-process#loc-peer-review-history)" target="_blank">what does this mean?</a>). If published, this will include your full peer review and any attached files.<br><br>

If you choose “no”, your identity will remain anonymous but your review may still be made public.<br><br>

<b>Do you want your identity to be public for this peer review?</b> For information about this choice, including consent withdrawal, please see our <a href="<https://www.plos.org/privacy-policy>" target="_blank">Privacy Policy</a>.

Reviewer #1: No

Reviewer #2: No

Reviewer #3: No

Figure Files:

While revising your submission, please upload your figure files to the Preflight Analysis and Conversion Engine (PACE) digital diagnostic tool, <https://pacev2.apexcovantage.com>. PACE helps ensure that figures meet PLOS requirements. To use PACE, you must first register as a user. Then, login and navigate to the UPLOAD tab, where you will find detailed instructions on how to use the tool. If you encounter any issues or have any questions when using PACE, please email us at [figures@plos.org](mailto:figures@plos.org).

Data Requirements:

Please note that, as a condition of publication, PLOS' data policy requires that you make available all data used to draw the conclusions outlined in your manuscript. Data must be deposited in an appropriate repository, included within the body of the manuscript, or uploaded as supporting information. This includes all numerical values that were used to generate graphs, histograms etc.. For an example see here: [http://www.plosbiology.org/article/info%3Adoi%2F10.1371%2Fjournal.pbio.1001908#s5](http://www.plosbiology.org/article/info%3Adoi%2F10.1371%2Fjournal.pbio.1001908#s5).

Reproducibility:

To enhance the reproducibility of your results, PLOS recommends that you deposit laboratory protocols in protocols.io, where a protocol can be assigned its own identifier (DOI) such that it can be cited independently in the future. For instructions see <https://journals.plos.org/plosntds/s/submission-guidelines#loc-methods>

__________________________________________________

In compliance with data protection regulations, you may request that we remove your personal registration details at any time. (Use the following URL: <https://www.editorialmanager.com/pntd/login.asp?a=r>). Please contact the publication office if you have any questions.

**REFERENCES:**

1.	Archer AM, Rico-Hesse R. High genetic divergence and recombination in Arenaviruses from the Americas. Virology. 2002;304(2):274-81.

2.	Charrel RN, de Lamballerie X, Emonet S. Phylogeny of the genus Arenavirus. Curr Opin Microbiol. 2008;11(4):362-8.

3.	Holzerland J, Leske A, Feneant L, Garcin D, Kolakofsky D, Groseth A. Complete genome sequence of Tacaribe virus. Arch Virol. 2020;165(8):1899-903.

4.	Ye C, de la Torre JC, Martinez-Sobrido L. Development of Reverse Genetics for the Prototype New World Mammarenavirus Tacaribe Virus. J Virol. 2020;94(19).

5.	Sayler KA, Barbet AF, Chamberlain C, Clapp WL, Alleman R, Loeb JC, et al. Isolation of Tacaribe virus, a Caribbean arenavirus, from host-seeking Amblyomma americanum ticks in Florida. PLoS One. 2014;9(12):e115769.

6.	Fedeli C, Moreno H, Kunz S. The Role of Receptor Tyrosine Kinases in Lassa Virus Cell Entry. Viruses. 2020;12(8).

7.	Hulseberg CE, Feneant L, Szymanska KM, White JM. Lamp1 Increases the Efficiency of Lassa Virus Infection by Promoting Fusion in Less Acidic Endosomal Compartments. mBio. 2018;9(1).

8.	Cohen-Dvashi H, Israeli H, Shani O, Katz A, Diskin R. Role of LAMP1 Binding and pH Sensing by the Spike Complex of Lassa Virus. J Virol. 2016;90(22):10329-38.

9.	Cohen-Dvashi H, Cohen N, Israeli H, Diskin R. Molecular Mechanism for LAMP1 Recognition by Lassa Virus. J Virol. 2015;89(15):7584-92.

10.	Carette JE, Raaben M, Wong AC, Herbert AS, Obernosterer G, Mulherkar N, et al. Ebola virus entry requires the cholesterol transporter Niemann-Pick C1. Nature. 2011;477(7364):340-3.

11.	Jae LT, Raaben M, Herbert AS, Kuehne AI, Wirchnianski AS, Soh TK, et al. Virus entry. Lassa virus entry requires a trigger-induced receptor switch. Science. 2014;344(6191):1506-10.

12.	Droniou-Bonzom ME, Reignier T, Oldenburg JE, Cox AU, Exline CM, Rathbun JY, et al. Substitutions in the glycoprotein (GP) of the Candid#1 vaccine strain of Junin virus increase dependence on human transferrin receptor 1 for entry and destabilize the metastable conformation of GP. J Virol. 2011;85(24):13457-62.

13.	Hallam SJ, Manning JT, Maruyama J, Seregin A, Huang C, Walker DH, et al. A single mutation (V64G) within the RING Domain of Z attenuates Junin virus. PLoS Negl Trop Dis. 2020;14(9):e0008555.

14.	Flanagan ML, Oldenburg J, Reignier T, Holt N, Hamilton GA, Martin VK, et al. New world clade B arenaviruses can use transferrin receptor 1 (TfR1)-dependent and -independent entry pathways, and glycoproteins from human pathogenic strains are associated with the use of TfR1. J Virol. 2008;82(2):938-48.

15.	Choe H, Jemielity S, Abraham J, Radoshitzky SR, Farzan M. Transferrin receptor 1 in the zoonosis and pathogenesis of New World hemorrhagic fever arenaviruses. Curr Opin Microbiol. 2011;14(4):476-82.

16.	Klewitz C, Klenk HD, ter Meulen J. Amino acids from both N-terminal hydrophobic regions of the Lassa virus envelope glycoprotein GP-2 are critical for pH-dependent membrane fusion and infectivity. Journal of General Virology. 2007;88:2320-8.

17.	Cosset FL, Marianneau P, Verney G, Gallais F, Tordo N, Pecheur EI, et al. Characterization of Lassa virus cell entry and neutralization with Lassa virus pseudoparticles. J Virol. 2009;83(7):3228-37.

18.	Sorkin A, Von Zastrow M. Signal transduction and endocytosis: close encounters of many kinds. Nat Rev Mol Cell Biol. 2002;3(8):600-14.

19.	Raaben M, Jae LT, Herbert AS, Kuehne AI, Stubbs SH, Chou YY, et al. NRP2 and CD63 Are Host Factors for Lujo Virus Cell Entry. Cell Host Microbe. 2017;22(5):688-96 e5.
