# Supplementary material for: A novel circulating tamiami mammarenavirus shows potential for zoonotic spillover
Source: PLoS Negl Trop Dis. 2020 Dec 28;14(12):e0009004. doi: 10.1371/journal.pntd.0009004 (PMC7794035; doi:10.1371/journal.pntd.0009004)
Supplement: Attachment — Submitted filename: Response to reviewers R3 PNTD.docx [file pntd.0009004.s014.docx]

We appreciate very much the constructive comments and scientifically enriching suggestions from all Reviewers. We have addressed all comments in the updated manuscript and we have answered below to each question separately.

**Reviewer's Responses to Questions**

**Key Review Criteria Required for Acceptance?**

**As you describe the new analyses required for acceptance, please consider the following**

**Methods**

-Are the objectives of the study clearly articulated with a clear testable hypothesis stated?

-Is the study design appropriate to address the stated objectives?

-Is the population clearly described and appropriate for the hypothesis being tested?

-Is the sample size sufficient to ensure adequate power to address the hypothesis being tested?

-Were correct statistical analysis used to support conclusions?

-Are there concerns about ethical or regulatory requirements being met?

**Reviewer #1:** -

**Reviewer #2:** My comments have all been addressed.

**Reviewer #3:** The authors have appropriately responded to my comments on the original submission.

--------------------

**Results**

-Does the analysis presented match the analysis plan?

-Are the results clearly and completely presented?

-Are the figures (Tables, Images) of sufficient quality for clarity?

**Reviewer #1:** -

**Reviewer #2:** My previous comments have been addressed. I have only a couple of minor additional comments on the results based on the revised version of the manuscript:

1) Fig S6B. The scale used here appears to be inappropriate for the data. It is impossible to estimate the increase between the very low values (below 1x107), while it is also necessary to break the scale at the top end to show the values for TAMV-REF D156N. This seems to be a clear indication that a log scale is needed here.

We agree with the reviewer, and consequently changed the scale of fig S6B to logarithmic.

2) Table 1 and S3. I do not believe the term “single nucleotide variant” is appropriate here as this refers to minority populations present within a quasispecies, whereas what the authors are analyzing here are consensus sequences for different species and/or isolates of a virus – as such it would be more appropriate to refer to these simply as “nucleotide changes”. Also it is confusing to show the nucleotide comparison data twice (on the top and bottom of the matrix) and may even be unintentionally misleading to some readers (since amino acid comparisons are usually shown on the bottom of such matrices). Fields that are not needed should simply be blacked out.

We share the opinion with the Reviewer and we have changed the tables 1 and S3. Following his/her suggestion, we have removed redundant data as well as changed the term “Single nucleotide Variant” for “Single Nucleotide Polymorphism”.

**Reviewer #3:** The authors have appropriately responded to my comments on the original submission.

--------------------

**Conclusions**

-Are the conclusions supported by the data presented?</br> -Are the limitations of analysis clearly described?</br> -Do the authors discuss how these data can be helpful to advance our understanding of the topic under study?</br> -Is public health relevance addressed?</br></br>

**Reviewer #1:** -

**Reviewer #2:** My previous comments have been addressed well. However, based on changes to the text, there a couple of additional points identified where the discussion may benefit from minor modification:

1) Lines 540-544. While it is true that arenaviruses are generally isolated primarily from a specific species, there is also evidence for evolutionary host switching among rodent species. Do the authors believe that mechanisms analogous to what they see here could also be involved in facilitating that process?

We appreciate very much the Reviewers comment and question. Indeed, not only the apparent promiscuity among Clade D mammarenaviruses but also the previously reported host switch events (1, 2), it is conceivable that similar mechanisms to those observed in our study also facilitate adaptation to new rodent hosts. We have commented on this in lines 540-1.

2) Lines 513-6. The authors suggest that a recently reported V64G mutation could be responsible for the attenuation seen in Candid#1, despite increased hTfR1 usage. However, while I agree with the authors that other mutations must be contributing to the Candid#1 phenotype, associating this with V64G specifically seems not to be well supported. In particular, this mutation results in “attenuation” (i.e. a growth deficit) when introduced into non-candid#1 JUNV strains (i.e. Romero) but does not do this for Candid#1 itself, which grow just as well as Romero does despite containing this mutation. This suggests that in the native context this mutation is compensated for by yet another layer of mutations. Rather, F427I has been shown to play a leading role in Candid#1 attenuation, with other mutations (e.g. T168A) possibly also contributing. Given our currently very limited knowledge on this issue, it is highly recommended that the authors limit themselves to referring to other changes in Candid#1 that affect its biology and could counterbalance the effect of hTfR1 and leave it at that to avoid overemphasizing specific mutations that in the end may not actually be relevant at all.

Following the Reviewer’s comment and suggestion, we have moderated our statements (Lines 513-5) to avoid misinterpretation of previous and present findings.

**Reviewer #3:** The authors have appropriately responded to my comments on the original submission.

--------------------

**Editorial and Data Presentation Modifications?**

Use this section for editorial suggestions as well as relatively minor modifications of existing data that would enhance clarity. If the only modifications needed are minor and/or editorial, you may wish to recommend “Minor Revision” or “Accept”.

**Reviewer #1**: -

**Reviewer #2:** My previous comments have been addressed. However, there are a lot of small mistakes in the text, particularly relating to the figure IDs, that need to be carefully fixed. Specific issues that were identified are listed below, but it is also necessary that the authors carefully check their text again for accuracy.

In addition to all the comments below, the authors have revised the manuscript to ensure accuracy and clarity.

1) Line 134. The phrase ‘utilizes hTfR1 to infect” is duplicated.

The mentioned sentence has been corrected (line 134).

2) Table 1. The authors should replace the reference to “fragment S” with “S segment” as this is the standard nomenclature. Please check the manuscript one more time to make sure this is corrected throughout.

We have verified the whole manuscript and corrected wrong references to genomic segments.

3) Line 976. Shouldn’t this be “L segment” instead of “S segment”?

We are thankful of Reviewer’s correction and we have change the mentioned line (977) accordingly.

4) Figure S2A and S4. Shouldn’t this be VSV M that is shown in this Western Blot? Compare text lines 209-211, 982-3, 1009.

In agreement with Reviewer’s comment, we have corrected figures S2A and S4.

5) Lines 988 and 991-992. These statements regarding the analysis of significance in the data are partly redundant.

We have corrected lines 989-90 to avoid unnecessary redundancies.

6) Lines 211-216. I believe the reference should be to Fig S2 not S1

The comment from the Reviewer is completely correct and the referenced figure is S2, not S1. We have modified the manuscript according to this.

7) Line 223, 234, 239, 900. Shouldn’t these statements refer to Fig S3 instead of Fig S1/S2?

The Reviewer is again correct and we have corrected aforementioned references.

8) Figure S3. The labelling in panel A is confusing as it contains no reference to being done in 293T cells (unlike all other panels in this figure, which specify the cell line used).

According the Reviewer’s comment, we have indicated the cell line (293T) in Figure S3A.

9) Line 295-8. This statement is not derived from the authors’ results and thus rather belong in the discussion.

In agreement with the Reviewer’s comment, we have moved the mentioned sentence to the discussion section (Line 492-5).

10) Line 928. Shouldn’t this be “…colored as in panel B”?

We have now corrected the mistake pointed by the Reviewer (line 929).

11) Line 360. This should be “293T and A549 cells….(Figs 5B, C, respectively)” since the data for 293T cells are presented first in Fig 5B.

Following the Reviewer’s comment, we have corrected the mentioned sentence (Line 357).

12) Line 940. This should be A549 cells not 293T cells.

For a clearer description of the experiments performed, as pointed out by the Reviewer, we have modified lines 938-44.

13) Line 943. This appears to be the description for panel E, a description of panel D is missing.

We have included now the panel description corresponding to panel D (lines 946-52).

14) Line 457. I believe this should be Fig 7B, C.

We appreciate the Reviewer’s comment and we have corrected the mentioned reference (line 454).

15) Line 471. A reference to the data in Fig 7D (for the mutants in the TAMV-REF background) is missing here.

As suggested by the Reviewer, we have now introduced a reference to the data presented in Fig 7D.

16) Line 590. Should this be “mapping analyses (Fig. 4)…”?

The reference to Fig 4 has been corrected (line 591).

17) There appears to be no reference to Text S1 within the manuscript. Also in Text S1, lines 7 and 9, I believe the authors are referring to the data shown in Fig S5 not Fig S2.

As indicated by the Reviewer, we have introduced a reference to Figure S5, which was neither referenced in the manuscript, as well as to Text S1 in lines 523-5, and corrected lines 7 and 9 in Text S1.

18) Text S2. Lines 8, 10 and 19. I believe the references should be to Fig S6 and not S3.

Again, the Reviewer is correct and we have corrected the references to Fig. S6 in Text S2.

**Reviewer #3:** (No Response)

--------------------

**Summary and General Comments**

Use this section to provide overall comments, discuss strengths/weaknesses of the study, novelty, significance, general execution and scholarship. You may also include additional comments for the author, including concerns about dual publication, research ethics, or publication ethics. If requesting major revision, please articulate the new experiments that are needed.

**Reviewer #1:** After addressing the comments of reviewer #2 the manuscript is further improved and I recommend accepting it.

We are very thankful of the Reviewer’s opinion of our work and his/her recommendation for its acceptance.

**Reviewer #2:** It needs to be ensured that the GenBank IDs for TCRV are added in the final version of the manuscript.

The Genbank’s accession numbers of segments L and S of TCRV-FL are MW150032.1 and MW150033.1 (Segments L and S, respectively), and are now indicated in lines 166-7.

We appreciate very much the comments, constructive criticism, and general evaluation of our study.

**Reviewer #3:** The authors have appropriately responded to my comments on the original submission.

We greatly appreciate the Reviewer’s comments and evaluation of our study.

--------------------

PLOS authors have the option to publish the peer review history of their article (<a href="<https://journals.plos.org/plosntds/s/editorial-and-peer-review-process#loc-peer-review-history>" target="_blank">what does this mean?</a>). If published, this will include your full peer review and any attached files.<br><br>

If you choose “no”, your identity will remain anonymous but your review may still be made public.<br><br>

<b>Do you want your identity to be public for this peer review?</b> For information about this choice, including consent withdrawal, please see our <a href="<https://www.plos.org/privacy-policy>" target="_blank">Privacy Policy</a>.

Reviewer #1: No

Reviewer #2: No

Reviewer #3: No

Figure Files:

While revising your submission, please upload your figure files to the Preflight Analysis and Conversion Engine (PACE) digital diagnostic tool, <https://pacev2.apexcovantage.com>. PACE helps ensure that figures meet PLOS requirements. To use PACE, you must first register as a user. Then, login and navigate to the UPLOAD tab, where you will find detailed instructions on how to use the tool. If you encounter any issues or have any questions when using PACE, please email us at [figures@plos.org](mailto:figures@plos.org).

Data Requirements:

Please note that, as a condition of publication, PLOS' data policy requires that you make available all data used to draw the conclusions outlined in your manuscript. Data must be deposited in an appropriate repository, included within the body of the manuscript, or uploaded as supporting information. This includes all numerical values that were used to generate graphs, histograms etc.. For an example see here: <http://www.plosbiology.org/article/info%3Adoi%2F10.1371%2Fjournal.pbio.1001908#s5>.

Reproducibility:

To enhance the reproducibility of your results, PLOS recommends that you deposit laboratory protocols in protocols.io, where a protocol can be assigned its own identifier (DOI) such that it can be cited independently in the future. For instructions see <http://journals.plos.org/plosntds/s/submission-guidelines#loc-materials-and-methods>

__________________________________________________

In compliance with data protection regulations, you may request that we remove your personal registration details at any time. (Use the following URL: <https://www.editorialmanager.com/pntd/login.asp?a=r>). Please contact the publication office if you have any questions.

**REFERENCES:**

1.	Fulhorst CF, Bennett SG, Milazzo ML, Murray HL, Jr., Webb JP, Jr., Cajimat MN, et al. Bear Canyon virus: an arenavirus naturally associated with the California mouse (Peromyscus californicus). Emerg Infect Dis. 2002;8(7):717-21.

2.	Cajimat MN, Milazzo ML, Hess BD, Rood MP, Fulhorst CF. Principal host relationships and evolutionary history of the North American arenaviruses. Virology. 2007;367(2):235-43.
